# Quantitative Determination of Cellular-and Neurite Motility Speed in Dense Cell Cultures

**DOI:** 10.3389/fninf.2019.00015

**Published:** 2019-03-12

**Authors:** Andreas W. Henkel, Lulwa A. A. D. Al-Abdullah, Mohammed S. Al-Qallaf, Zoran B. Redzic

**Affiliations:** Department of Physiology, Faculty of Medicine, Kuwait University, Kuwait City, Kuwait

**Keywords:** cell motility, velocity measurement, image analysis, membrane contraction, pericytes, hippocampal neurons

## Abstract

Mobility quantification of single cells and cellular processes in dense cultures is a challenge, because single cell tracking is impossible. We developed a software for cell structure segmentation and implemented 2 algorithms to measure motility speed. Complex algorithms were tested to separate cells and cellular components, an important prerequisite for the acquisition of meaningful motility data. Plasma membrane segmentation was performed to measure membrane contraction dynamics and organelle trafficking. The discriminative performance and sensitivity of the algorithms were tested on different cell types and calibrated on computer-simulated cells to obtain absolute values for cellular velocity. Both motility algorithms had advantages in different experimental setups, depending on the complexity of the cellular movement. The correlation algorithm (COPRAMove) performed best under most tested conditions and appeared less sensitive to variable cell densities, brightness and focus changes than the differentiation algorithm (DiffMove). In summary, our software can be used successfully to analyze and quantify cellular and subcellular movements in dense cell cultures.

## Introduction

Analysis of changes in cellular motility, shape changes and movements of subcellular particles plays an important role in exploring cell biology phenomena. Video imaging of cells *in vitro* is commonly used to analyze these processes. Cells show often highly dynamic morphological changes and large translocations after application of drugs and chemicals that affect the cytoskeleton or organelle trafficking inside the cytoplasm (Paluch et al., 2005; Krause and Gautreau, 2014). Though these morphodynamic effects are very obvious upon visual inspection, they could be difficult to quantify, because few software tools exist that could measure nonlinear movements of cellular objects and structures (Myers, 2012; Barry et al., 2015). The existing programs we found so far, do all require dye-stained preparation and cannot be used in low- quality phase contrast images without major manual intervention to select the structures of interest (Rodriguez et al., 2008; Jacquemet et al., 2017; Urbancic et al., 2017).

One strategy, addressing this problem was the development of particle image velocimetry (PIV) (Vig et al., 2016). It has widely been used for motion analysis from cytoplasm streaming during embryonal development (Brangwynne et al., 2009), quantification of bacterial flow (Dombrowski et al., 2004) and dynamics of the cytoskeleton in migrating cells (Ponti et al., 2004). The approach assumes that sufficiently large areas of the visual field stay close together, similar to floating rafts, which restricts usefulness of this approach to *in vitro* cultures where individual cells moved collectively. Additionally, further correction algorithms were necessary to compensate for compromised images with a low signal-to-noise ratio (Vig et al., 2016).

In most cell cultures cellular and subcellular movements occur randomly and cellular processes or cells overlap. Non-directional movements of cells and their processes could often cancel each other out. Therefore, we employed a strategy, where single components were digitally separated and then analyzed individually, assigning these individual components into clearly defined object classes. This task required the development of algorithms that could sort these structures into classes, based on their morphological characteristics. In order to obtain absolute mobility values, digital simulations of moving cells were employed where the artificial objects closely resembled the originals with regard to size, form and movement characteristics. The motility of the simulated objects was set by user-defined parameters to correlate very close to the real cell movements and calibrated these values to the original data by linear functions in order to obtain absolute motility velocities.

We developed a software that enables quantification of several aspects of cellular dynamics under conditions where individual objects could not be singled out sufficiently. The rationale behind this approach was to measure global mobility changes of specific object classes in image series. This was achieved either by separating well-defined structures (e.g., cell membranes, processes, or small globular particles) from raw images and measuring the brightness-distribution differences between successive frames (Differential Movement = DiffMove algorithm) or by determination of a correlation coefficient between image frames and its correction by image ratio calculation (Combined Pearson's Correlation and Ratio Analysis Movement = COPRAMove algorithm). The two algorithms were implemented in the image analysis software “SynoQuant,” which was developed and programmed by AWH within the framework of a large image analysis package from SynoSoft.

This approach was applied to several cell cultures types, which were maintained for up to 48 h in an incubation microscope and images were taken at regular time intervals. Primary cultures of hippocampal cells (Henkel et al., 2010), which were composed of a mixture of glial cells and neurons with sprouting neurites (Welzel et al., 2010), chicken-telencephalon-derived glial cells, which were used to study the movement of intracellularly organelles, and primary cultures of rat brain pericytes (Yemisci et al., 2009), which are large spider-shaped cells that can contract or relax their cellular processes spontaneously or in response to drugs and could change membrane dynamics upon deprivation from oxygen or drug treatment (Hill et al., 2014).

The obtained data suggest that both algorithms had advantages in different experimental setups, depending of the complexity of the cellular movement, but the correlation algorithm (COPRAMove) performed better under most tested conditions because it appeared less sensitive to variable cell densities, brightness and focus changes.

## Materials and Methods

### Animals

Primary cultures of pericytes were produced from 1 to 2 months old male or female Sprague Dawley rat weighting 200–220 g. Hippocampal neuronal cultures were prepared from newborn Wistar rats. Animals were supplied by the Animal Resource Center (ARC), Health Science Centre (HSC), Kuwait University. All experiments were carried out in accordance with the guidelines of laboratory animal care in HSC and the protocols were approved by ARC. Fertilized White Leghorn chicken eggs were obtained from the institutional animal care facility University Erlangen, and all procedures using animals were approved by the Institutional Animal Care and Use Committee.

### Cell Cultures

#### Hippocampal Cultures

Hippocampal neurons of 1- to 3-day-old Wistar rats were isolated and cultured according to a previous protocol with minor modifications (Klingauf et al., 1998; Henkel et al., 2010). In brief, 2–3 newborn rats per experiment were sacrificed by cervical dislocation, both hippocampi were removed from the brain and immersed into ice-cold Hank's salt solution (HBSS). After digestion with trypsin (5 mg/ml), cells were triturated mechanically and plated in modified Eagle's medium (MEM), supplemented with 10% fetal calf serum and 2% B27 supplement (all from Invitrogen, Germany) on 18 mm glass coverslips, coated with poly-L-lysine (PLL) (Sigma). The coverslips were placed in 12- well plates and cells were used after 5 days in culture. Cells treated with ketamine (Sigma), end concentration of 1 μM, received the drug acutely, and were then taken immediately to the microscope.

#### Primary Culture of Rat Brain Pericytes

We used protocol described by Yemisci et al. (2009) that was modified as explained earlier (Redzic et al., 2015). Briefly, rats were anesthetized with urethane (1 g/kg) and sacrificed by cervical dislocation, brains were removed and placed in ice-cold Ca^2+^ and Mg^2+^ free HBSS, (Gibco), 10 mM HEPES and 0.6% BSA (both Sigma) and adjusted to pH = 7.3. After cleaning of associated tissues, the brain was sliced, and digested by 1-h incubation in collagenase/dispase solution (2,000 Kunitz units/mL) that contain serine proteases inhibitor tosyl-L-lysyl-chloromethane hydrochloride (0.147 mg/ml) in HBSS, pH = 7.3. Microvessels were separated as a pellet from the homogenate by centrifugation at 1,490 × g for 15 min, washed twice in HBSS and then incubated in collagenase/dispase solution for 2.5–3 h. At the end, digested microvessels were seeded into flasks (25 cm^2^ surface) that were pre-coated with rat tail collagen in Dulbecco's Modified Eagle Medium (DMEM) that contained 20% (v/v) fetal bovine serum, 5 μg/ml vitamin C (all from Sigma) and 1% (v/v) antibiotic/antimycotic solution. The cultures were washed with PBS and the medium replaced after 48-h and every 2–3 days thereafter. Cultures were passaged after 7–10 days to uncoated flasks in order to eliminate endothelial cells. Cultures were used for experiments 4–5 days after the passage.

For oxygen glucose deprivation (OGD) experiments, flasks were transferred to an anoxia glove box (Plas BY Labs-Lansing, MI. USA) with in inner atmosphere consisting of 5% CO_2_ and 5% H_2_ in N_2_ and incubated for up to 6-h in glucose and pyruvate—free DMEM (Gibco, UK). A palladium catalyst (Plas BY Labs-Lansing, MI, USA) was used to remove any residual oxygen. Anaerobic conditions were confirmed with anaerobic strip indicators before and during the time course of incubation, following instructions by the manufacturer (Oxoid, Hampshire, UK). All media and buffers that were used for OGD experiments were maintained inside the glove box for at least 12 h prior to the experiments in order to equilibrate pressure of gasses.

#### Chicken Telencephalon Cell Culture

Telencephalon cortices were dissected from E14 chicken embryos (4–6 brains/experiment) and cells were isolated and cultured as described earlier (Pettmann et al., 1979). Cultures were grown at 37°C in a humidified atmosphere of 95% air and 5% CO_2_, and experiments were performed at the fifth day *in vitro* (Henkel et al., 2006).

### Cell Culture Imaging

Two days old hippocampal cultures (30,000 cells/cm^2^) were kept in 25 cm^2^ closed PLL-coated culture flasks, and clamped on a 2D movable holder into a temperature controlled chamber on the phase-contrast microscope (“Cell Observer,” Zeiss, Germany). The microscope was mounted on an anti-vibration air table (Newport, USA). The focus was visually controlled and adjusted (every 8–12 h) since the autofocus system was not working reliably at the magnification through a 10 × objective. Images were collected every 15 min over a period of 2 days.

Twelve days old primary cultures of pericytes were transferred to the “Cell Observer” incubation microscope and images were taken every 5 min for 15 h. The higher frequency of imaging and shorter observation period were chosen to better resolve fast cellular motility. Images were taken only from areas that did not contain confluent cell layer or obviously overlapping cells, which permitted individual cells to be digitally excised from the original image frame and analyzed separately.

Imaging of telencephalon chicken cells was performed on an Axiovert 135 microscope (Zeiss) and images were collected every 2 s to observe intracellular trafficking of organelles, stained with acridine orange (100 nM, Molecular Probes, Eugene, USA). For application of staurosporine (0.1–1 μM, Calbiochem, San Diego, USA) during the experiment, image acquisition was stopped for 1 min, the drug was carefully added by perfusion and image acquisition was resumed at the same recording frequency.

### Image Analysis

#### Preprocessing of Images

Images were initially stored in uncompressed Audio Video Interleave (AVI) video format, transferred to an external computer, converted to lossless images in BMP format and systematically organized in folders to ease automated images analysis. Transfer, extraction and batch processing was carried out by SynoQuant software. Since many image series suffered from uneven illumination and low contrast, SynoQuant was used to correct and standardize the images. As a first step, all images were resized to 640 × 480-pixel size and converted from 24 bit RGB color to 8-bitmap gray-scale images (brightness range: 0–255). Images were then normalized to a mean brightness level of 127-pixel brightness, using SynoQuant's build-in “Center bright” function. “Center bright” calculated symmetric brightness distribution of the pixel values in a histogram. Morphometric analysis required an additional processing step to compensate uneven illumination. Therefore, the background was corrected with the function “Flatten Background” to obtain a uniform brightness distribution.

#### Image Processing Algorithms

Several image processing algorithms were employed to standardize the images in order to compare image series between each other and remove artificial image defects that could compromise the cell mobility analysis. Fully automated batch processing allowed a high throughput of several folders containing the images series. All SynoSoft programs (~190 MB), including SynoQuant, and the complete Visual Basic code for all procedures and algorithms can be downloaded from:

https://www.dropbox.com/sh/y7b78jwrzvooyz1/AAB4yV4X1P4ByDOtoPv66RDHa?dl=0
The zipped setup file: SynoSoft_Setup.7z can also directly downloaded at https://www.dropbox.com/s/a9l7zpq5d3a0vih/SynoSoft_Setup.7z?dl=0
An overview on the program package is shown at www.synosoft.de.

#### Sharpen Images

Images were sharpened to amplify cells and processes shapes. There were several algorithms available in SynoQuant's repertoire but the most common used methods were removal of hazy areas from the original image by local subtraction of a Gaussian blur from bright center pixels or employing the digital “Unsharp mask” algorithm (Ferrari et al., 2009).

#### Particle Removal

Cell culture images were very often contaminated with small debris particles that were removed by the “Remove debris particle” algorithm. The particles were detected by a contrast- based function and further defined by their small size. Their positions were finally filled with a highly blurred local background that had the same brightness as the neighborhood.

#### Background Normalization

The extended “Subtract background image” algorithm generated a brightness equalization (flattening) combined with smooth background. All stationary structures were removed by subtraction of a brightness-scaled mean image that was composed of all images in the series.

#### Focus Determination

The focus of images changed sometimes during the course of video imaging. Since unsharp images could severely affect subsequent quantitative mobility determination, the focus was measured according to the following procedure: First brightness gradients were processed with a Laplacian edge detector, resulting in an image with enhanced differences between neighboring pixels. The degree of image sharpness (*F*) was quantified by calculating the logarithm of the squared total brightness standard deviation (*sd*) of this image.

$$F=ln\left(sd^{2}\right)-1$$

The range of *F* was between ~-1 (homogenously blurred flat image) and ~8 (binary image with clear edges). Optionally, the software could automatically remove all sections when the focus quality felt below a preset threshold.

#### Structure Processing for Individual Cell Analysis

**Figure 1:**
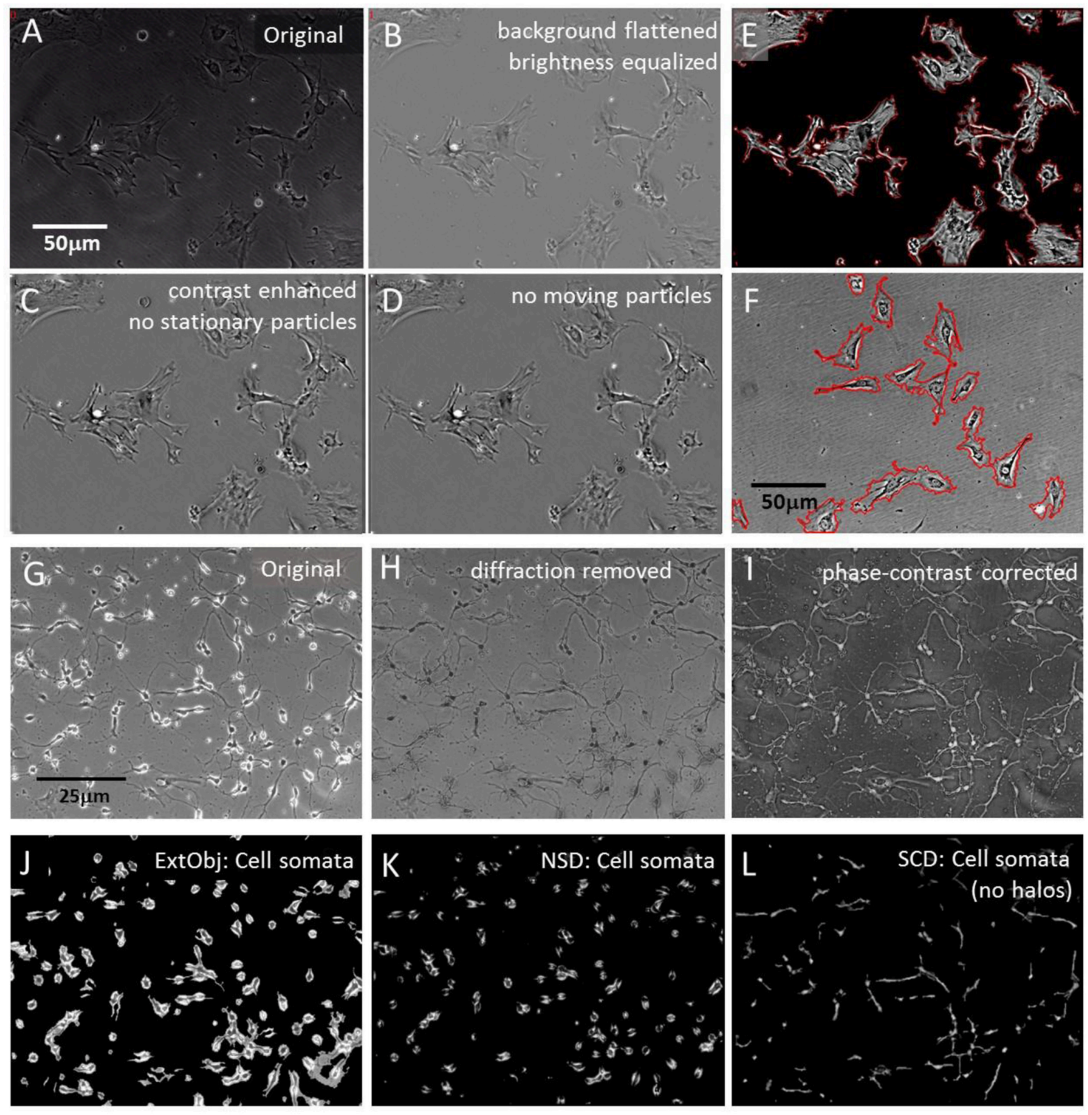
Segmentation algorithms. **(A)** Original raw image of cultured pericytes; **(B)** raw image processing: brightness normalization and background equalization; **(C)** grayscale contrast enhancement and stationary particle removal; **(D)** removal of mobile particles; **(E)** plasma membrane detection with “Statistical Cell Detection” and subsequent enhancement with red mask; **(F)** set of pericytes, processed with brightness normalization and background equalization but no contrast enhancement. Plasma membrane detection with “Isolate cells from background” and subsequent enhancement with red mask; **(G)** Original raw image of hippocampal cells, 3 days in culture; **(H)** raw image processing: light diffraction halos around somata removed by “Level light diffraction” algorithm; **(I)** raw image processing: pseudoDIC effect and brightness inversion subjected to “Phase-contrast correction” algorithm; **(J)** segmentation of hippocampal cells with the “Extract bright objects” algorithm; **(K)** segmentation of hippocampal cells with the “Neuronal somata detector” algorithm; **(L)** segmentation of “Phase-contrast corrected” hippocampal cells with the “Statistical Cell Detection” algorithm.

Motility quantification of plasma- and intra-cellular membranes in large cells, like pericytes, required standardization, brightness normalization and cell segmentation to conduct cell-to-cell comparison of morphodynamics parameters. Initially, a brightness equilibration, combined with a background flattening was performed; cells were contrast-enhanced and small stationary debris particles were removed. Finally, moving debris particles were removed too and stationary cells (5–10 per series), that showed membrane ruffling and organelle trafficking but no large-scale translocation were manually labeled and automatically segmented for individual motility and membrane dynamic analysis. Circular label masks were set spaciously onto the individual cells to provide enough room for expansion and constriction. The cells were automatically sorted into separate folders and membrane ruffling velocity was measured with the COPRAMove algorithm. Figures 1A–D show the results of these processing steps.

#### Segmentation of Plasma Membranes

Quantification of cell constriction and expansion required a segmentation of the perimeter, and exclusion of internal organelles, cytoskeleton and membranes. Cell perimeters were segmented by two algorithms. The first, “Statistical Cell Detection” (SCD), used a statistical approach to select similar groups of pixel intensities the corresponded to specific cellular structures. It selected brightness- and area clusters in brightness histograms that had a higher frequency than a user defined threshold and connected adjacent pixels to each other. In a second stage, only objects were selected that exceeded a minimal size and small spiky appendages were removed. The output of this algorithm were grayscale images that contained only the cells and had a plain background. The cell perimeters were than labeled with a line mask and only these structures (corresponding to plasma membranes) were analyzed by motility detection algorithms. Figure 1E shows an example of labeled membranes.

An alternative algorithm “Isolate cells from background” (ICB) used another principle to detect cell perimeters. Its detection was based on contrast differences between the background and the cell borders and on and object size range. Since this mechanism also labeled high contrasted intracellular structures, it was followed by a completion-fill function that identified all enclosed structures and leveled them. The result of was a homogenous mask that covered the whole cell area. Figure 1F shows the perimeter of the mask, overlaid onto the original image.

#### Removal of Light Diffraction

Phase contrast images often contained bright diffraction light halos around cells that were not fully attached to the surface (Figure 1G). The halos were a specific marker for loosely attached neuronal soma but were not desirable if neurite movements were quantified, since they contributed over-proportionally to general motility. Therefore, these artifacts were removed by SynoSoft's algorithm “Level diffraction light” in the “Reduce diffraction light (Phase contrast)” menu. First, the 5% brightest pixels were selected, low pass filtered and their intensity was reduced by 50%. The resulting image was stored and subtracted from the original image. In a second step, the intensity histogram of the original image was normalized to 1 and each pixel was multiplied with the reciprocal of its respective intensity distribution.

$$intensity_{\text{new}}=intensity*\frac{1}{\left((Histogram\left(intensity\right)*255)+1\right)}$$

Finally, the new image was merged with first processed image and inverted, resulting in a diffraction-free picture (Figure 1H). Some image series were further enhanced by inversion and shaded contrasting, using the “Correct phase contrast” algorithm to obtain a pseudo differential interference contrast (pDIC) image (Figure 1I).

#### Cell Soma Segmentation

Neuronal cells consist of a soma and sprouting neurites. In order to study the motility of cell somata and neurites separately, the two structures were segmented and analyzed independently. Cell somata extraction was achieved by three algorithms, where their application depended on the image quality and experimental design. The first algorithm “Extract bright objects” (ExtObj) used diffraction halos around non-tightly attached neurons as soma marker (Figure 1J). It detected brightness peaks that surpassed a local percentile threshold. Single neighboring pixels were connected, holes in cell bodies filled and small debris particles removed. This algorithm was the fastest of all soma-detector algorithms.

Two alternative algorithms were used to detect neuronal somata in unprocessed images The “Neuronal somata detector” (NSD) algorithm used also diffraction halos to detect somata (Figure 1K). The detection principle was based on high contrast differences around halo regions. Smaller high-contrast debris artifacts were subsequently removed and the halos were reduced by subtraction of a blurred image of these regions. Finally, the image was brightness-corrected to compensate the loss of intensity due to the subtraction operation.

The “Statistical Cell Detector” (SCD), removed elongated thin structures and background areas from the image by selection of conjugated brightness- and area clusters in intensity histograms. It performed better if the images were enhanced with the “Correct phase contrast” algorithm prior to its application. SCD selected only pixels in the brightness histogram that had a higher frequency than a user defined threshold. In a second stage, only objects were selected that exceeded a minimal size and small spiky appendages were removed. Figure 1L shows the result of this procedure, applied to the image in Figure 1I.

#### Neurite Segmentation

**Figure 2:**
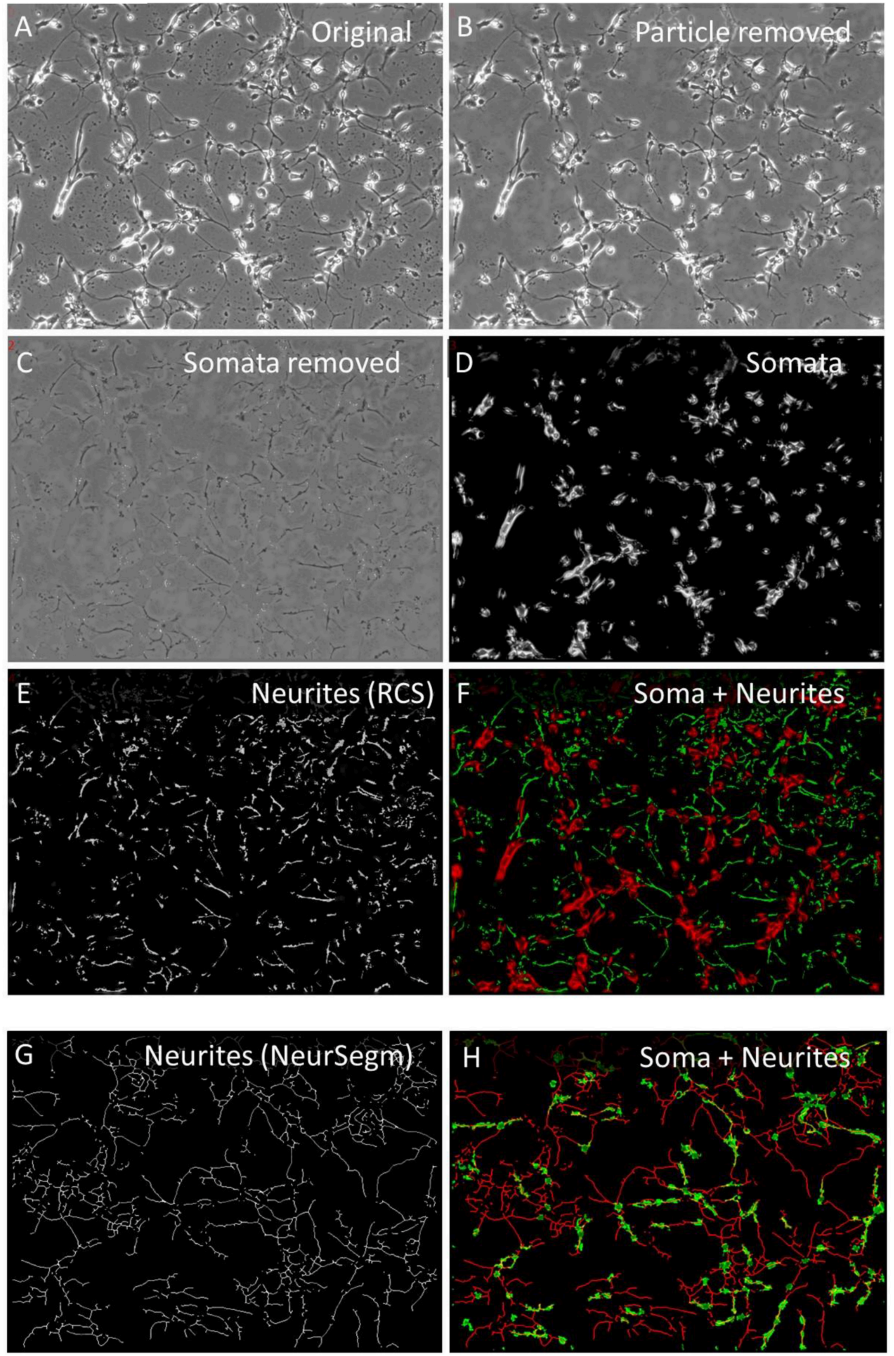
Neurite segmentation with “Remove cell somata” (RCS) algorithm. **(A)** Original raw image of hippocampal cells, 2 days in culture; **(B)** contaminating particles removed by “Remove debris particle” algorithm; **(C)** RCS: somata removed, **(D)** somata obtained with the “Extract bright objects” algorithm from image **(B)**; **(E)** inverted and background subtracted neurites from image C; **(F)** overlay image of somata (red) and neurites (green); **(G)** skeletonized neurites, segmented with “Trace and Quantify” module; **(H)** overlay image of neurites frim image **(G)** (red) and somata (green), segmented with “Neuronal somata detector” algorithm.

Neurites were segmented to study their soma-independent mobility by two multi-procedural algorithms. The “Remove cell somata” function (RCS) identified large areas that had a lower local contrast that the neuronal somata, which were characterized by bright halos. Figure 2A shows the original image and Figure 2B the first step of this procedure that started with the removal of small debris particle. The low—contrast areas were protected while the high contrast areas became excised. The position of the cell somata were filled with background and smoothed to blend into the immediate environment (Figure 2C). Figure 2D shows the somata as they were detected in parallel by the “ExtObj”—function. Figure 2E shows the inverted and background-subtracted neurites from Figure 2C as they were used as input for both motility quantification algorithms. The separation of somata and neurites was complete as shown in Figure 2F, which displays the colored overlay of neurites and somata.

Neurites were also segmented by the “Trace and Quantify” module. This algorithm (NeurSegm) generated a binary skeleton of the neurites. At first, it selected dark elongated structures and removed cell somata and background areas, defined by local contrast, local threshold and size classification. Figure 2G shows only neurite skeletons, segmented from the original image Figures 2A,H visualizes the complete separation of somata and neurites in an overlay image.

### Analysis of Cell Motility

Motility analysis was conducted with build in algorithms of “SynoQuant” and results were compared to particle tracking results, obtained from the Fiji-ImageJ2 (Rueden et al., 2017) plugin “TrackMate” (Tinevez et al., 2017). The software worked independent from the cell species, because it measured differences and correlations between successive whole images. However, since different cell types exhibited different motility velocities, it was advisable to acquire images of fast moving cells at higher frequencies, because “SynoQuant” had no lower limit for detecting slow cell motility.

#### Definition of Movement

Different movement categories were defined to characterize several types of cellular mobility (van Larebeke et al., 1992). The term “Motility” included any form of active cell movement. “Translocation” specified movement of the cell soma on the substrate while “ruffling” described formation, retraction, or any type of plasma membrane movement, including sheet-like pseudopodia and cytopodia (Bracke et al., 1991). “Trafficking” was defined as any movement of intracellular objects, including lysosomes, transport vesicle, nuclei, Golgi apparatus, granules, etc.

#### Correlation-Based Motility Quantification

Statistical correlation between images was performed by measuring their relative similarity of the spatial brightness distribution. SynoQuant implemented a correlation-based algorithm, “P-move,' to measure cell motility speed by calculation of a Pearson's correlation coefficient between successive images. An advanced modification of the correlation algorithm was adopted by the “COPRAMove” algorithm that included an image-ratio method to compensate focus-change related artifacts and used a set of digitally simulated cells to calibrate the absolute translocation speed of cell somata between successive images in [pixel/frame]. The “COPRAMove” function was only slightly affected by variable cell numbers, focus changes and robust against brightness fluctuations.

**Figure 3:**
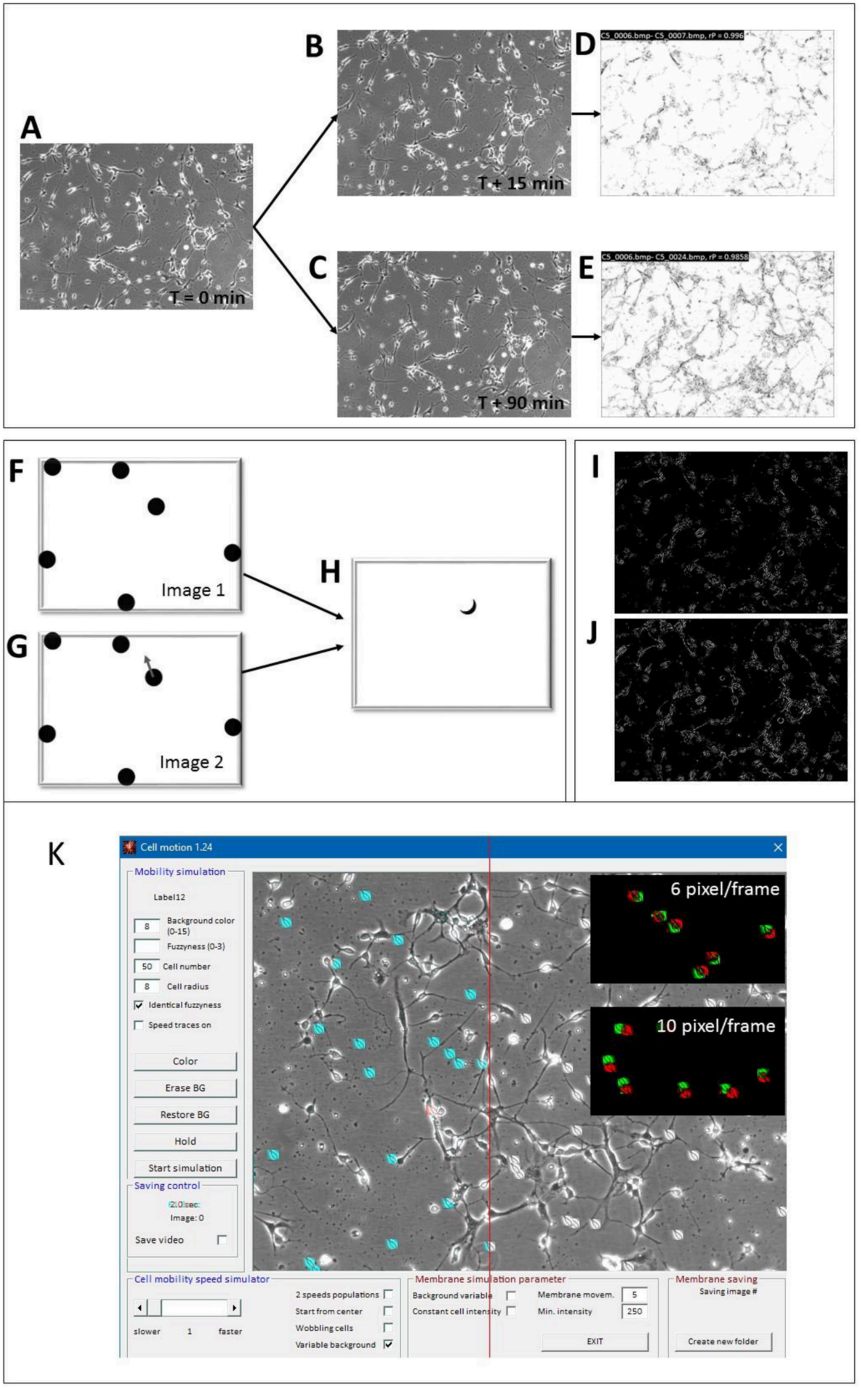
Motility detector algorithms and “Cell simulator” test module. **(A–E)** Principle of COPRAMove algorithm; **(A)** Original image of hippocampal cells at the start (t = 0) of the experiment, **(B)** image acquired after 15 min; **(C)** image acquired after 90 min; **(D)** scaled correlation image between images **(A,B)**; **(E)** scaled correlation image between images **(A,C)**. **(F–H)** Principle of “DiffMove” algorithm; **(F)** schematic simulated cells; **(G)** schematic simulated cells with one cell shifted; **(H)** difference image F and G shows partially shifted cell section. **(I–J)** Principle of ratio corrector algorithm; **(I)** scaled ratio image between 2 a dwell time of 15 min time difference; **(J)** scaled ratio image between 2 images with a dwell time of 90 min time difference. **(K)** Screenshot of “Cell simulator” module: 50 simulated cells are overlaid onto a background image of a hippocampal cell culture preparation. Twenty-one cells, left from the red line are colored in cyan for better visibility, upper black inset: overlay image of 2 consecutive frames, assigned to red and green channels, respectively, where cell translocation speed was set to 6 pixels/frame, lower black inset: overlay image of 2 consecutive frames, assigned to red and green channels, respectively, where cell translocation speed was set to 10 pixels/frame.

Prior to the analysis, all images were size-standardized to either 640 × 480 or 512 × 512 pixels to allow absolute speed calibration. The algorithm consisted of a two-step process. At first, subsequent images were mean-filtered (3 × 3 matrix) for noise reduction and a Pearson's correlation coefficient (rP) was calculated between successive images. Figures 3A–E visualizes the effect of cellular movement by correlating 2 images that were taken at *T* = 15 min (Figure 3B) and at *T* = 90 min (Figure 3C) with respect to the start image at *T* = 0. The local correlation appeared to be high (white areas in the correlation image in Figure 3D), if no or only minor movement had occurred while, high cell motility speed corresponded to a proportionally lower correlation, shown in Figure 3E. Then the images were subjected to a Sobel edge-detection filter (Uppala and Sahr, 1997) operation and a ratio between successive images was calculated. Ratios that surpassed a user-defined threshold were counted by a linear increment of the ratio factor (*rF*). The ratio speed operator (*rSO*) was calculated as the square root of the product of inverse image size × *rF* × 20 and scaled up by a factor of 10:

$$rSO=\sqrt{\left(\frac{1}{img\;size*rF*20}\right)}*\;10$$

Finally, the COPRA motility descriptor (COPRAMove) that corresponded to the relative motility of the cells, was calculated from the *rP* and *rSO*, according to the formula:

$$COPRAmove=\frac{\left(1-rP\right)*100}{rSO}$$

The absolute mean translocation of cells [pixels/frame] was then calculated by means of SynoQuant's “CellSimulator” module, using a linear transform function, obtained from a set of simulated cells, moving at different defined velocities.

#### Difference-Based Motility Quantification

Any active cellular motility resulted in position and/or shape changes and these differences corresponded to local brightness variations. The magnitude of this difference corresponded to the relative displacement of cells. Figures 3F–H visualizes a simplified principle of the measurement under ideal conditions. If one cell changed its position (cell with arrow in image 2, Figure 3G), the difference image, resulting from the subtraction of the second from the first image, showed a difference area (Figure 3H), which was principally proportional to the translocation velocity of the cell. The faster the cell moved, the larger was the difference area in the subtraction image. The “DiffMove” algorithm is described as follows:

Successive images were subtracted from each other and the number of pixels that changed their brightness was calculated. Whenever there was a brightness change that surpassed a preset threshold, the pixel was counted. The threshold was defined to avoid that random noise could significantly contribute to the movement measurement. It was set to a brightness level of 10, in all experiment series.

The motility descriptor “DiffMove” of the difference algorithm, corresponding to the relative movement of cells, was derived from dividing the number of pixels, surpassing the threshold, by the total number of non-black pixels, multiplied by a scaling factor of 10,000.

$$absolute\;difference=\sum_{\text{x}1,y1}^{\text{xn},yn}\left|img1-img2\right||$$

If the absolute difference between corresponding pixel intensities surpassed a user-defined threshold in successive image frames, the number of valid difference pixels (Z) was incremented.

Z=Z+1

Finally, the sum of Z corresponded to the total mobility of cells in the image pair. To obtain a normalized mobility score for an image pair, the following formula was used:

$$Diffmove=\frac{\text{Z}}{\text{N}(\text{pixel})\text{*}10000}$$

“DiffMove” was defined as the relative cell motility in the difference algorithm.

#### Ratio-Based Mobility Measurement Assistance Algorithm

The brightness ratio between consecutive time laps images in a series corresponds to their relative differences. In order to measure the degree of object translocation, edge detection of image structures was performed and the ratio between successive images was calculated. This procedure reduced random noise from images and focused on the motility of larger highly contrasted objects like cells and neurites. Finally, a user defined threshold was set. A low threshold (<10) included small changes between successive image frames, while a high threshold (>30) emphasized the movement of fast objects. Figure 3I shows the ratio image of two consecutive frames (15 min dwell time), while Figure 3J shows the ratio image between 6 frames (90 min dwell time). Based on the procedural strategy, the algorithm was sensitive to focus change, brightness fluctuation and structure density.

#### Simulation of Cell Soma Motility

Simulation of moving cell-like objects allowed the determination of absolute cellular translocation speed in an images series, which was used for quantitative analysis and calibration of the mobility algorithms. Cell somata were either modeled as ellipsoids with a variable shape, diameter and fuzziness or images of real cells were used as moving object sprites. Figure 3K shows the SynoQuant's simulation module “CellSimulator” user interface with images of real cells (highlighted in blue), moving over a real image of hippocampal neurons. “CellSimulator” controlled motility speed, cell size, cell shape, membrane movement (wobbling, ruffling), background noise, cell brightness and cell number. Simulated cells or images of real cells could be overlaid onto a static background image to simulate a realistic environment. The red-green images (insets) demonstrate two different translocation velocities that could be visualized by assigning successive frames with moving cells to red and green channels. The colored images showed larger distances between the centers of faster moving cells (10 pixels/frame) compared to slower moving cells (6 pixel/frame). The simulator generated several sweeps of 50 images each, where the cells moved over an original background picture. Each sweep corresponded to a defined mean translocation speed of the digital cells, expressed in [pixels/frame]. The image series were then quantified by the motility analysis algorithms and linear correlated to the motility descriptor results from COPRAMove and DiffMove algorithms.

Two parameters were defined to describe the dynamic activity of the cell membrane. First, the ratio between cell area and cell perimeter: $APr=\frac{Cellarea}{Cellperimeter}$ and second the cell membrane contractility (CtraM). CtrM was defined as the dynamic change in cell area according to the formula:

For each frame [i], containing one single cell:

$$CtrM=\sqrt{\frac{{\left(pA_{i}-pA_{i-1}\right)}^{2}}{{\left(pA_{i}+pA_{i-1}\right)}^{2}}}\;$$

where pA is:

$$pA=\frac{Cell\;area_{i}}{Maximal\;cell\;area}*100$$

### Statistics

Statistical analysis was performed with SynoStat 1.22 (SynoSoft) and Microsoft Excel 2013 (Microsoft). Kolmogorov-Smirnov test and Shapiro-Wilks test were used to check if the data followed a normal distribution. ANOVA test was used to check if differences between experimental groups were larger than intra-group variations. Normally distributed data were tested for significant differences between groups with Student's *t*-test, while a non-parametric data distribution was analyzed with Wilcoxon-Mann-Whitney *U*-test. The significance acceptance level was set to *p* < 0.05 in both tests. To assess the effect size of an experimental intervention, Cohen's D was calculated to compare experimental groups. The effects size was determined to: 0.2 to 0.3 “small,” around 0.5 “medium,” > 0.8 “large” (Cohen, 1988). Pearson's and Spearman's correlation coefficients for assessment of mutual dependencies were calculated with SynoQuant's build-in module “Histogram and Correlation Plot Analysis.”

## Results

### Visual and Quantitative Simulation of Cell Motility With “Cellsimulator”

**Figure 4:**
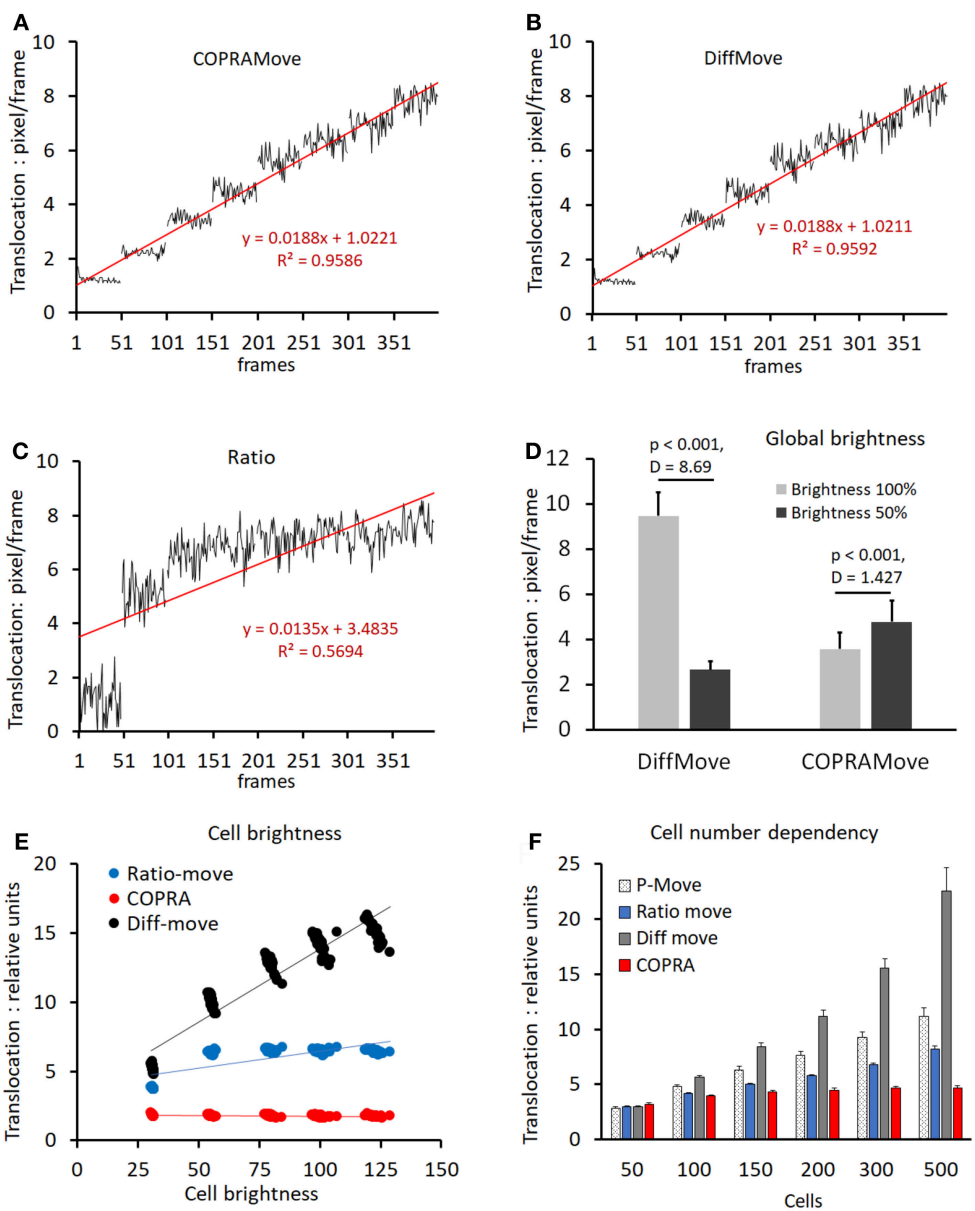
Motility velocity measured on simulated cells. **(A)** translocation velocity measured with “COPRAMove” algorithm on 8 image sweeps, 50 frames each, of simulated moving cells (n = 200), increasing their mean velocity linearly by 1 pixel per step; **(B)** translocation velocity measured with “DiffMove” algorithm on the same set of images as in **(A)**; **(C)** translocation velocity measured with “Ratio” algorithm on the same set of images as in **(A)**; **(D)** motility algorithms sensitivity to global brightness changes (mean and standard deviation); **(E)** motility algorithms sensitivity to cellular brightness changes (mean and standard deviation); **(F)** motility algorithms sensitivity to cell number changes (mean and standard deviation).

Verification and quantitative performance of the motility algorithms were analyzed by means of cell motility simulations, conducted by SynoQuant's movement simulator module “CellSimulator.” Since the translocation velocity of randomly moving cells was directly defined in units of [pixels/frame], a linear function could be derived to calibrate the COPRAMove velocity descriptor in SynoQuant's mobility analysis module. Figures 4A–C show calibrated translocation traces, derived from both tested motility algorithms, and the ratio correction algorithm, including their linear transfer functions and corresponding *R*^2^ correlation coefficients (see insets). Each step represented a sweep of 50 frames. It is noteworthy to point out that the ratio algorithm is an integral part of the COPRA algorithm, and functions as a compensator for focus changes.

The translocation velocity of digital cells was stepwise increased by 1 pixel/frame, generating 8 sweeps that were subjected to quantification. The “COPRAMove” and the “DiffMove” algorithms (Figures 4A,B) showed an approximately linear correlation at increasing translocation velocities, but also a trend to level-off and becoming less discriminative in the higher velocity range. Their random noise increased also at higher speed. The ratio algorithm in Figure 4C showed a fast increase at very low velocity but was not able to quantitatively describe the translocation of faster moving cells.

Besides velocity, “CellSimulator” controlled individual cell-brightness and a global intensity level of the image; therefore, it was employed to test, if a global brightness affected the translocation measurement results of the algorithms. The total image intensity was reduced to 50% by and the translocation of the original and the darker image series were quantified. Figure 4D showed that translocation, measured with the “DiffMove” algorithm was significantly 3.5-fold decreased, while COPRA response was 0.75-fold higher in darker images. Besides testing effects of a global brightness variation, we changed the brightness of individual simulated cells. Figure 4E showed that a progressive cell-associated brightness increase had a strong effect on the translocation results, obtained with “DiffMove” algorithm. The translocation motility values correlated closely to the cellular brightness (*R*^2^ = 0.893). Translocation measurements with the “Ratio-move” algorithm where generally independent from cell brightness (*R*^2^ = 0.548), except for the transition at low light levels. COPRAMove's results were largely independent from an increasing cellular intensity (*R*^2^ = 0.2067).

Finally, the effect of a variable cell number on translocation results was analyzed. Changing cell numbers are commonly observed in cell lines with a high rate of mitosis or in cultures, where cells migrate substantially, leading to cluster formation or disintegration. Figure 4F showed that an increasingly higher number of cells, moving constantly at a speed of 4 [pixels/frame], exhibited a significantly higher translocation as measured by all algorithms. The extent of the increase varied considerably among the algorithms. The “COPRAMove” algorithm increased the apparent relative translocation only 1.1-fold, when the number of cells was raised from 100 to 500. The “DiffMove” (increase 3.9-fold), the “Ratio-move” (increase 1.9) and the basic Pearson's correlation “P-move” (increase 2.3-fold) algorithms showed much stronger effects. The results of all algorithms were statistically significant elevated when the lowest cell density, *n* = 50 was compared to the highest, *n* = 500 cells. The relative cell-number-independent performance of the COPRAMove algorithm, which was composed of ratio and “P-move” algorithms, was attributed to a mutual compensation between these both components.

### Organelle Motility in Fluorescently Stained Cells

**Figure 5:**
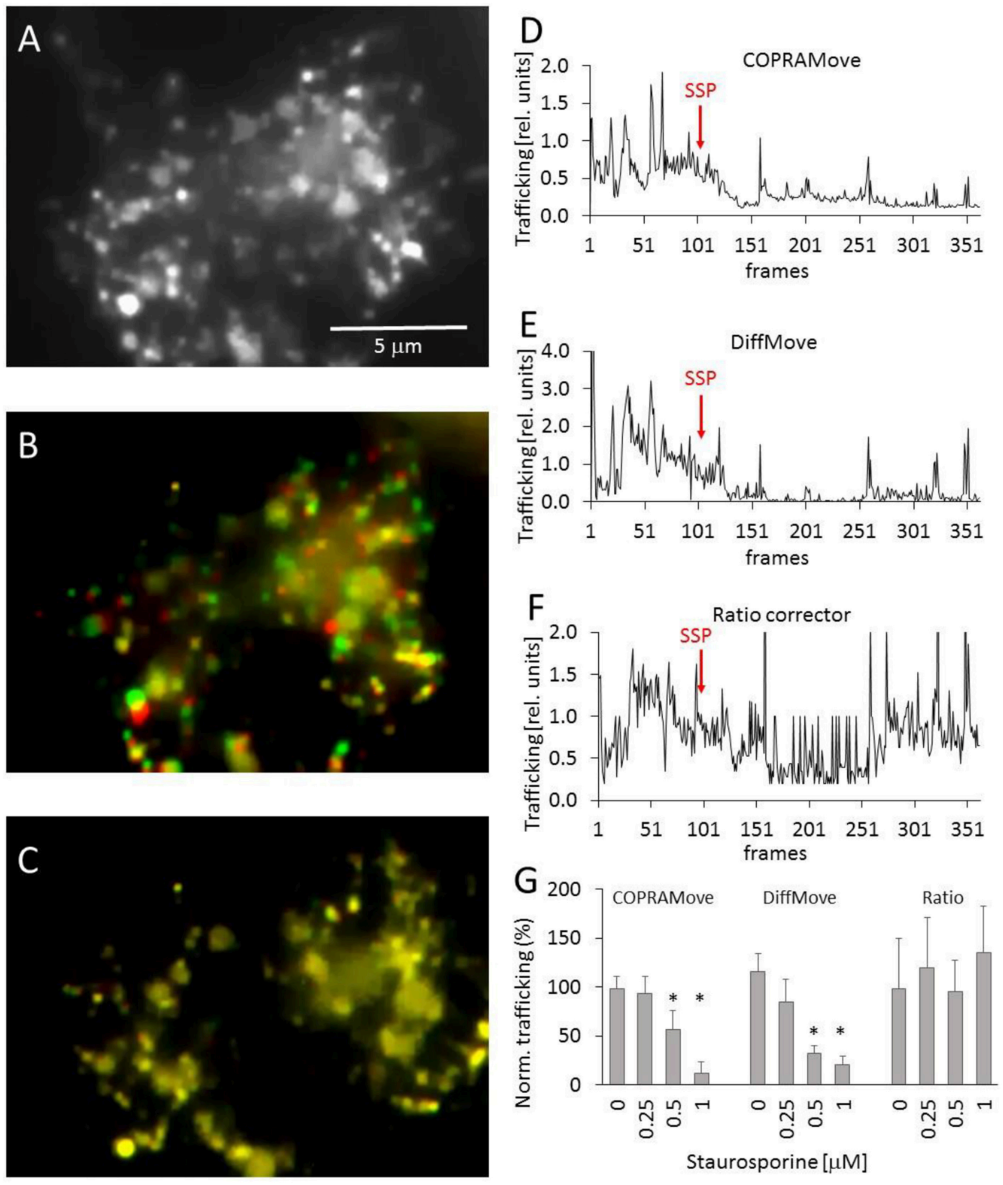
Vesicle motility in chicken telencephalon glia cells. **(A)** Fluorescence image of acidic organelles in a glia cell, labeled with acridine orange; **(B)** overlay image of two consecutive frames, frame 40 (red) and 41 (green) before addition of staurosporine; **(C)** overlay image frame 300 (red) and 301 (green) after addition of staurosporine; **(D)** vesicle trafficking velocity trace, recorded with “COPRAMove” algorithm; **(E)** vesicle trafficking velocity trace, recorded with “DiffMove” algorithm; **(F)** vesicle trafficking velocity trace, recorded with Ratio corrector algorithm; **(G)** Vesicle motility reduction by staurosporine (mean and standard deviation, asterisks indicate significant difference to control, p < 0.05).

Chicken telencephalon cell preparations are composed of several cell types, including neurons and glial cells (Mangoura et al., 1995). Large, flattened glia cells were selected to measure intracellular trafficking velocity of acidic organelles, fluorescently labeled with acridine orange (Figure 5A) in time laps videos. The apparently random movements of lysosomes and vesicles was determined during a control period to obtain a baseline trafficking value. The velocity of the organelles was slowed down by addition of staurosporine, a drug that reduced vesicle movement in the frog neuromuscular junctions and chromaffin cells (Betz and Henkel, 1994; Henkel et al., 2000). The Supplementary Video 1 showed the dynamic of the organelle movement. Figure 5B visualized the vesicle traffic in an overlay image that was composed of two consecutive images, assigned to different color channels. The two images were collected during the initial phase of the experiment, before any drug was added. The first image was assigned to the green and the second to the red color channel and overlaid, providing an optical visualization of dynamic organelle trafficking. Background areas and objects without substantial trafficking appeared in yellow, while objects in red or green indicated a pronounced organelle velocity. Figure 5C showed that the addition of staurosporine significantly (*p* < 0.01) slowed down the movement of all organelles, indicated by the predominant yellow color. Since there were several degrees of the color overlays, it was very difficult to quantify the overall trafficking velocity of the organelles by just measuring the apparent brightness of the two color channels.

The organelle trafficking in the image series was then quantified by the algorithms “COPRAMove,” “DiffMove,” and “Ratio-move.” This experimental setup (fluorescently stained preparation) was chosen as a test example for a class of images with a dark background and bright moving objects. Figures 5D,E showed that the “COPRAMove” and the “DiffMove” algorithms mimicked the subjective visual impression of organelle trafficking very close, while the “Ratio-move” algorithm (Figure 5F) did not reflect the apparent vesicle trafficking very well. Figure 5G showed that increasing concentrations of staurosporine inhibited organelle trafficking dose-dependent.

### Motility Measurements in Hippocampal Cultures

**Figure 6:**
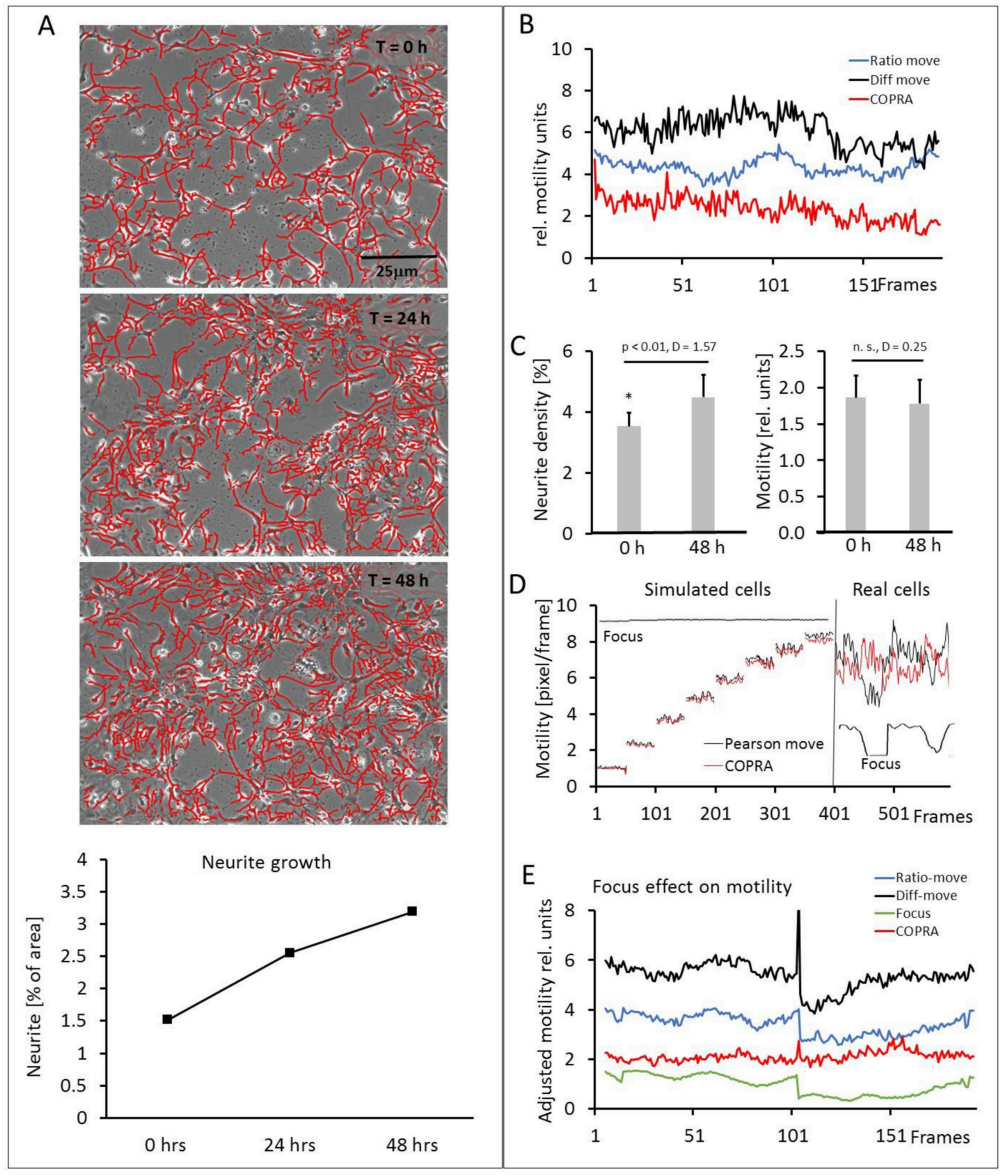
Motility measurements on hippocampal neurons. **(A)** Visual presentation of a neurite network development (3 upper) and its quantification (lower). Neurites are masked with “Trace and quantify” module; **(B)** exemplary motility velocity traces without soma-neurite separation; **(C)** quantification of neurite density and motility changes within 48 h; **(D)** focus dependency of motility recordings in simulated and real hippocampal cells; **(E)** Comparison of motility speed and focus dependency.

Our hippocampal cultures consisted of neuronal and glia cells (10 image series, 152.6 ± 32.3 cell somata per frame). The neurons developed an increasingly denser network of neurites within the course of the 48 h experiment, but their number did not increase. Figure 6A and the video (Supplementary Video 2) showed a scenario of the neurite development over 48 h. The neurons, glia cells and neurites moved in an apparently random process and this overall motility was quantified in detail. Figure 6B shows examples of three motility traces, measured in a typical experiment. The “COPRAMove” and the “DiffMove” algorithms indicated that the general motility decreased slightly in this time laps series, despite a large increase in neurite number and density. Motility, determined with the “Ratio-move” algorithm indicated no general trend but rather a wave-like time course that mimicked the focus trace (data not shown). The mean motility, averaged from 10 time lapse series (Figure 6C) confirmed that cellular motility speed decreased a little, but not significantly within the observation period, while neurites formed an extended clearly visible dense network.

Then we investigated the effect of image sharpness on motility measurements, because there were frequent temporary focus changes observed during a 48-h long imaging experiment. The “COPRAMove” algorithm was examined in particular, because it included an image correlation- and an image ratio algorithm. The performance of a simple Pearson's correlation- based algorithm (“P-move”) without any subsequent corrective functions and the “COPRAMove” algorithm were compared on simulated- and real cells. Figure 6D showed that “COPRAMove” and “P-move” performed very similar on simulated cells that moved with increasing velocities, but “P-move” could not compensate temporary focus variations in real life experiments and its motility trace resembled very much the course of the focus trace. “COPRAMove” motility measurements were apparently not affected by fading focus.

To further investigate the phenomenon on motility measurement, an image series was chosen that had a large step-like focus change. Cellular motility together with the global image focus were measured. Figure 6E showed that the Ratio-corrector algorithm, and to a lesser extent the “DiffMove” algorithms were very sensitive to focus changes too, since their motility traces followed largely the focus trace. The “COPRAMove” algorithm appeared to have no correlation with focus fluctuations, because of the compensatory effect its inverse ratio-correction term.

**Figure 7:**
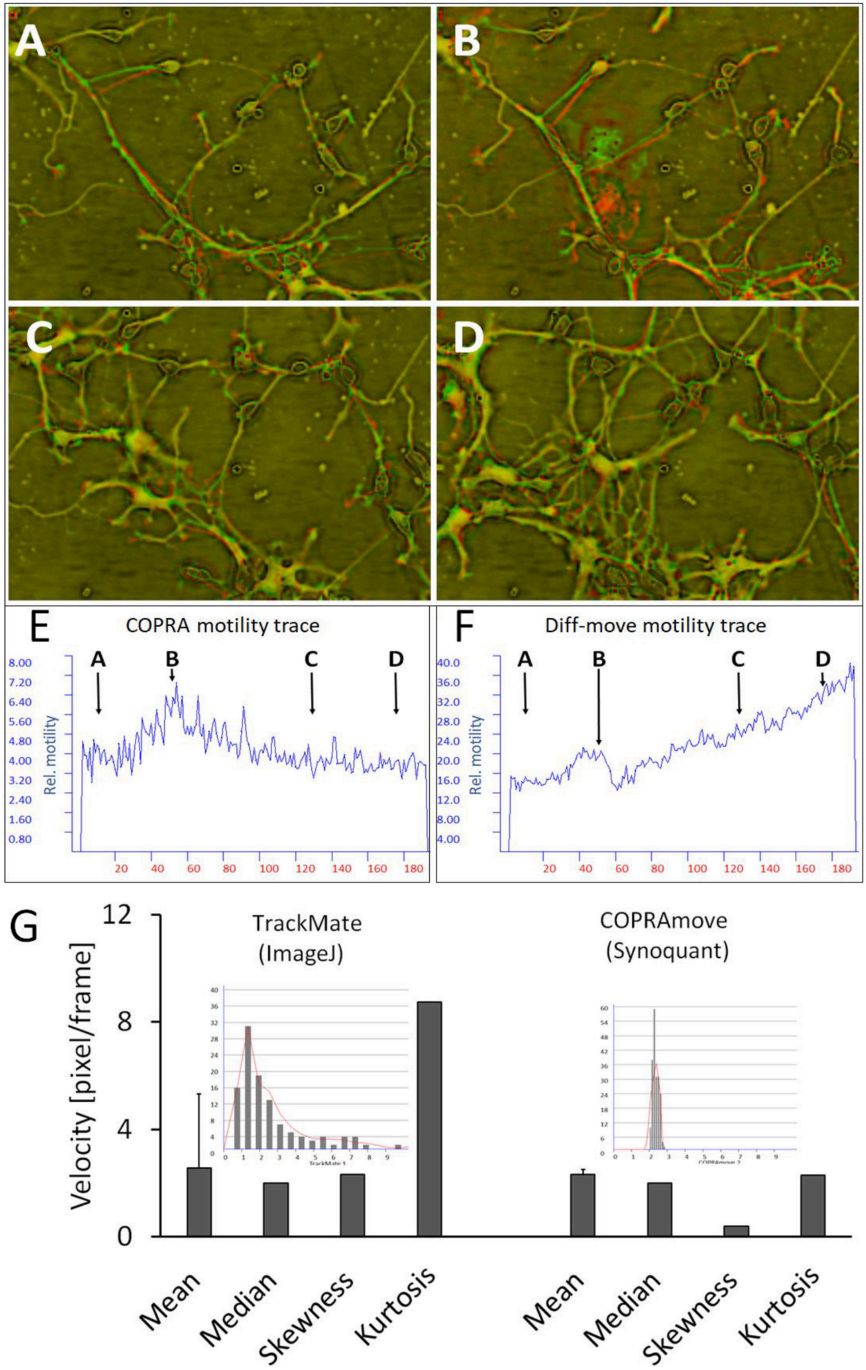
Motility measurement on soma-neurite networks with increasing density. **(A–D)** Successive image frames, assigned to red and green channels, visualize neurite movements and growth of neurite network at different time points. **(A)** at 2.5 h; **(B)** at 12.5 h; **(C)** at 32.5 h; **(D)** at 44 h; **(E)** COPRAMove motility velocity trace; **(F)** DiffMove motility velocity trace; **(G)** Comparison of velocity distributions by COPRAmove and TrackMate (ImageJ plugin). The insets are histograms of velocity measurements, obtained by the respective algorithms. TrackMate automatically tracked mean velocities of 113 cells over 20 frames, COPRAmove measured the velocities of segmented cell somata in a series of 192 frames.

The effect of variable cell number on motility quantification, which was observed in the study with simulated cells, was checked in hippocampal neuron-glia co-cultures. These preparations were composed of a variety of neuron- and glia cell types, among them microglia. These immune cells moved with extremely high speed, compared to all other cells, and were a good tool to judge the performance of motility algorithms (McGlade-McCulloh et al., 1989). We chose a field of view that contained a high dynamic range of cell numbers and -motility, including temporary appearance of microglia cells. The Supplementary Video 3 provided a visual impression of the dynamic activity. Figures 7A–D clarified the cellular translocation and shape changes by means of consecutive red-green overlay-images, where pure red and green colors indicated high local motility. Two motility traces, measured by “DiffMove” and “COPRAMove,” emphasized the busy cellular motion in Figure 7E. These traces provided quantitative data of the visual impression that the general movement velocity stayed generally constant despite an increase of structure count. At begin, the view field was sparsely populated but as the neurite network started to grow, a very fast moving microglia cell (Figure 7B) was temporary swirling over the scene. The view field was constantly filled with invading cells and a growing neurite network (Figures 7C,D), which moved at an apparent steady velocity. “COPRAMove” and “DiffMove” traces showed a transient motility increase in the presence of the fast moving microglia cell, but the “DiffMove's” motility trace continued to rise, due to the increasing number of cluster-forming cells. COPRA's motility trace returned to the initial level, indicating a constant motility speed.

### Comparison of Cell Somata Motility Quantification by Tracking and Copramove Algorithm

Direct tracking of cells is an establish method to obtain motility data from individual cells. The ImageJ plugin “TrackMate” is an advanced software that detects and measures the speed of roundish cells by an automated algorithm. “COPRAMove” velocity results of cell somata were compared to “TrackMate” on a series of 192 images of hippocampal cells. “TrackMate” detected 112 cells in a frame and recorded their positions and velocities. The program lost increasingly more cells during the course of the series, as they changed their shape and intensities. The resulting individual velocities were plotted in a histogram (inset in Figure 7G) that showed a heavily right side—skewed distribution with a mode of 1.2 pixel/frame. The mean value of 2.6 ± 2.8 s. d. and the median of 2 pixels/frame were in the same order of magnitude like the velocity data, which were obtained by “SynoQuant.” Figure 7, right panel, showed that the velocity distribution over the course of the image series did not vary much, but was also skewed toward the right with a 5.9-times lower skewness and a 3.8 times lower kurtosis.

### Somata and Neurite Analysis

**Figure 8:**
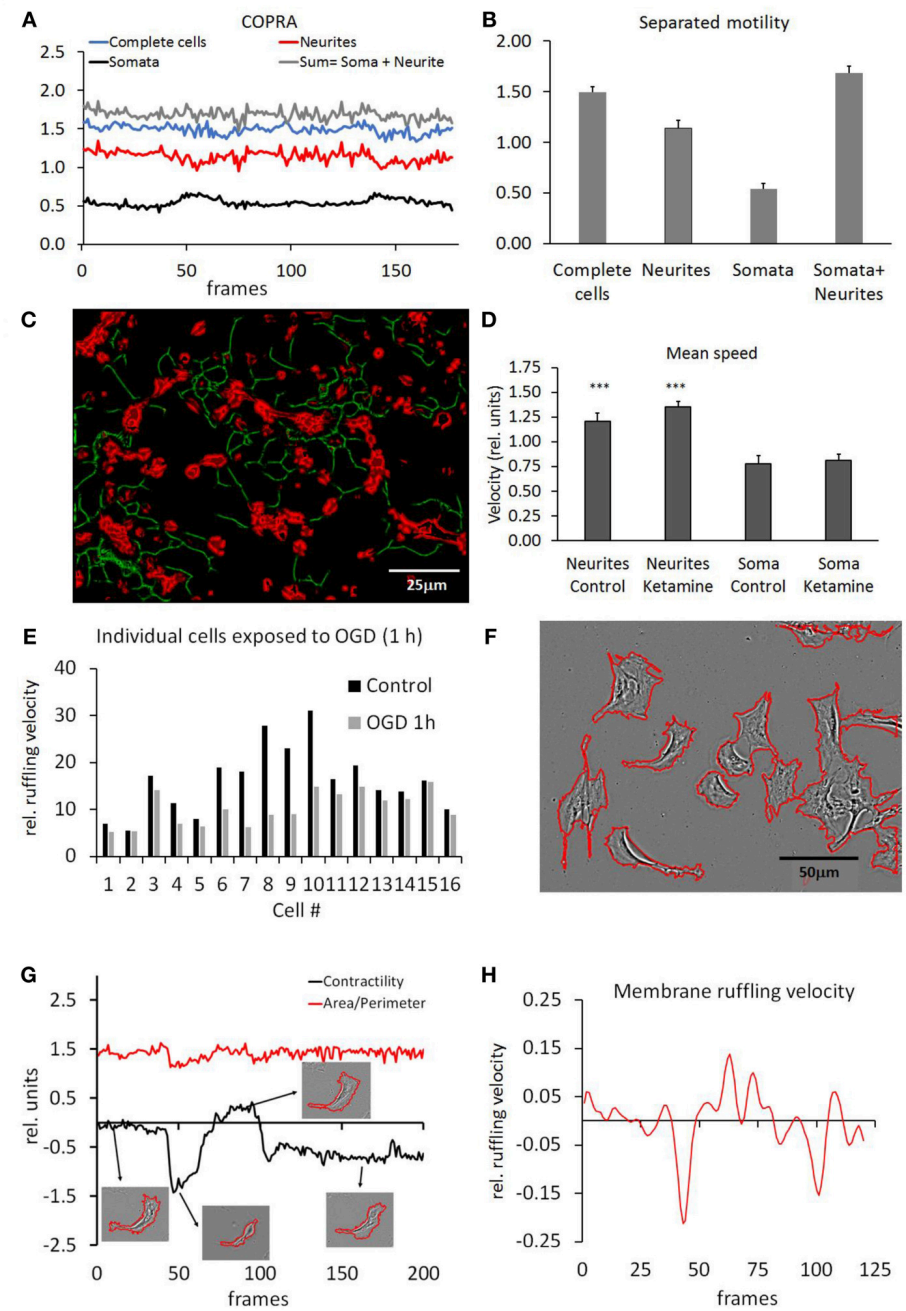
Motility measurements on segmented structures. **(A)** COPRAMove motility traces, obtained from isolated somata, neurites and combination of both. “Complete cells” refers to unsegmented hippocampal cultures, “Somata + Neurites” refers to the summed traces of segmented neurites plus segmented somata; **(B)** Quantification of motility result; **(C)** representative image of segmented neurites (green) and somata (red). Motility speed of both components was measured separately by COPRAMove algorithm; **(D)** results of motility speed measurements of segmented neurons and somata (mean motility speed ± standard deviation, n = 11 experiments). Neurites are significantly faster after ketamine application. Somata speed does not change.; **(E)** plasma membrane ruffling velocity of pericytes, measured on isolated cell perimeters; **(F)** Pericyte perimeter outlined by automatic plasma membrane segmentation with the “Isolate cells from background” (ICB) algorithm; **(G)** contractility and area/perimeter traces show alternation contraction and extension of typical pericyte plasma membrane. The 4 insets show segmented cell perimeters, acquired at corresponding times (arrows) of the experiment; **(H)** moving average of membrane velocity trace, calculated with the “Trace and Quantify” module. The velocity corresponds to the 1st derivative of contractility trace in **(E)**.

Selective extraction of somata and neurites (segmentation) allowed the separated determination of their motility. We have tested three somata (SCD, NSD and ExtObj) and two neurites (RCS, and NeurSegm) segmentation algorithms. All algorithms detected the cell somata and neurites consistently but the detection probability was focus dependent. A weaker focus resulted in a lower detection probability and hence increased random noise in the motility traces. The NeurSegm algorithm was more sensitive than the RCS algorithm but this problem could be alleviated by averaging of successive frames. The RCS algorithm proved to be more selective for thicker neurites that were not easily lost upon a variable focus and therefore exhibited a lower noise level. Figure 8A shows three traces that were recorded from a typical time-lapse series. The first trace was recorded from unprocessed non-segmented hippocampal cells and motility was analyzed with the COPRAMove algorithm. The motility velocity did generally not change during the course of the experiment. This was consistent with the overall result in the set of 11 experiment series, which showed no statistically significant change in motility (Mean/SD: 1.88 ± 0.29, *n* = 11). The absolute motility values depended on the setting of the COPRAMove motion detection system and were only consistent when all experiment series were measured with the unchanged parameter. Absolute values required a calibration with the “CellSimulator” module.

Then neurites and somata were measured separately and their motility velocities were compared to the unprocessed series. The visual impression that the neurites moved slower than the somata, was confirmed by the COPRAMove quantitative analysis. Figures 8A,B show that the neurite trace had a lower mean velocity than the somata trace. If both traces were summed up, they were slightly larger than the trace, obtained from the non-segmented cells (somata with neurites). Differences between all four traces were statistically significant. The Supplementary Video 4 shows a colored overlay of separated somata and neurites. The images were averaged before the generation of the video to provide a better visual impression of the dynamic process.

Finally, we tested the COPRAMove algorithm on a large image dataset of 2 experimental series obtained from hippocampal mixed neuron and glia-cell preparations. We compared the motility speed of untreated control cells (*n* = 11 independent experiments) to cells that were treated with 1 μM Ketamine (*n* = 10). Somata and neurites were segmented (Figure 8C) and measured them separately to quantify the visual impression that ketamine accelerated the neuronal network motility speed. Figure 8D shows that there was no significant effect on the motility of segmented cell somata (*t*-test: n. s., D_Cohen_ = 0.35) but the digitally isolated neurite network moved significantly (*t*-test: *p* < 0.001, D_Cohen_ = 2.065) faster after ketamine application compared to untreated controls.

### Pericyte Motility

Pericytes were grown at low density that allowed subsequent analysis on individual cell basis (6–8 image series, 5.76 ± 2.27 cell somata per frame). Their individual motility speed was measured with the “DiffMove” algorithm, before and after they were subjected to an oxygen and glucose deprivation (OGD). Figure 8E shows that the motility of 16 pericytes was consistently and significantly (*t*-test: *p* < 0.01, D_Cohen_ = 1.069) decreased after 1-h of OGD, a condition that is known to reduce migration in human pericytes (Chen et al., 2015; Schneider et al., 2015).

### Dynamic Plasma Membrane Ruffling

The motility of large highly magnified pericytes was examined to quantify membrane ruffling, cell constriction and pseudopodia extrusion. Stationary pericytes were chosen to exclude cell translocation from the ruffling speed measurements. Figure 8F shows that the plasma membrane was segmented by means of the ICB algorithm; single cells were segmented and separately analyzed. The cells showed alternating periods of extension and retraction of pseudopodia and membrane extrusions under control conditions. We measured two parameters, cell area/perimeter ratio (APr) and membrane contractility (CtrM), which were used to describe this activity. We obtained a mean APr to characterize the steady-state roughness of the cell membrane which was 8.3 ± 0.76 sd, *n* = 25 cells. Six-hour exposure to reduced oxygen-glucose conditions caused a significant increase (*p* < 0.001) of the APr to 10.8 ± 0.73 sd, *n* = 17. A higher AP ratio indicated that the pericytes had fewer membrane protrusions compared to controls. The change of APr was rather slow (data not shown) and the plasma membrane ruffling speed, measured with the “DiffMove” algorithm, was slightly but not significantly increased (11.5 %, ± 25% sd, *n* = 16) over the course of the experiment. Then we analyzed the dynamic of plasma membrane changes, defined as contractility (CtrM). This parameter quantified the change of the 2-dimensional cell area in contrast to membrane roughness that was measured by the APr parameter. Under control conditions, the membrane exhibited sudden motility changes between extension and retraction that corresponded closely to the CtrM parameter, while the AP ratio indicated an overall largely constant cell shape. Figure 8G compares the contractility dynamic and the cell membrane shape (APr) on a typical pericyte. The shape of the cell did not show many or large protrusions, but the area size changed suddenly to a great extent. Since the cell volume couldn't change back and for so dramatically, as suggested by the images and the membrane ruffling velocity (Figure 8H), it was assumed that the cell was extending in height, invisible to the phase contrast microscope.

## Discussion

This study addressed two major challenges for efficient analysis of cellular morphodynamics, namely the segmentation of whole cells, cell membranes and cellular elements and secondly, their velocity analysis in densely grown cell cultures and cellular neuronal networks. We have tackled the problem of segmentation by the development of different procedures, based on independent mathematical methods that provided optimized solutions to almost any specific set of cells. A major challenge for these algorithms was to cope with images that have been acquired under more or less sub-optimal conditions (Dow et al., 1987). A human observer may easily distinguish a cell from other structures or debris, because he identifies the structures by a neuronal network, his brain, which integrates shape, contrast and brightness into a holistic picture (Spoerer et al., 2017). But even after successful object recognition, it is still very difficult and time consuming to quantify identified parameters manually or with help of semi-automated software tools. SynoQuant works largely fully automated after initial setting of detection parameters. In this study we separated cellular components into 3 categories: (1) Cell somata, (2) Cell appendages (e.g., neurites, growth cones, processes etc.), and (3) Plasma membranes.

In order to detect neuronal somata, most studies relayed on some form of labeling for soma detection (Kayasandik and Labate, 2016) or used confocal microscopy for this task (Ozcan et al., 2015).

### Segmentation of Somata

Segmentation of complex neurons with dendrites is a challenge that has been successfully addressed on fluorescent individual cells at high magnification. Morphological parameters in such cells were quantified to characterize morphodynamics changes on individual neurons (Billeci et al., 2013). Phase-contrast images at low magnification however, contained very different intensity distributions that are uncorrelated to cell boundaries and intracellular structures. Neurons showed usually a light diffraction halo around the soma membrane in phase contrast images, because they are not tightly attached to the substrate (Li and Kanade, 2009; Yin et al., 2012). This feature could be exploited to segment them from flatted glia cells and neurites, which both appeared to be much darker (Kandel et al., 2018). However, a complete separation of glia and neuronal cells was not possible without specific labeling, because some glia cell types showed also halos around their soma. Two algorithms, ExtObj and NSD, obtained soma segmentation by identifying their center positions by the corresponding halos and processed these regions different to extract alternative structures from it. ExtObj did not separate individual cells but rather cell clusters, enclosed in halos. NSD preferred the halo centers, isolating mostly individual cell somata. Subsequent velocity measurements revealed that both algorithms did not differ much regarding the motility results. One problem, however was that both algorithms, particularly ExtObj, did not consistently detect the somata boundaries always at the same positions in consecutive images, causing a flickering effect that added noise (~15% of the signal in both “COPRAMove” and “DiffMove”) to the segmented image series. NSD was marginally more consisted in “COPRAMove” motility analysis but this had only little effect on the overall velocity results (~12% of the signal “COPRAMove,” 18% in “DiffMove”). The responsible factor was mainly a locally variable focus due to vertical cell movement that mainly contributed to the problem. The SCD algorithm segmented very specifically the somata when halos were removed by preprocessing with the “Phase-Contrast-Correction” algorithm, but the principal local focus sensitivity remained still a problem. In summary, all three segmentation algorithms were similar suited for velocity measurements, even if the visual impression of the segmented cells appeared different to the human eye.

### Segmentation of Neurites

Segmentation of neurites in hippocampal cultures have been obtained by several groups on highly resolved neurons to analyze neurite development and 3D structures with conventional algorithms (Meijering, 2010; de Santos-Sierra et al., 2015) or convolution neuronal network (CNN) software implementations (Kandaswamy et al., 2013; Li et al., 2017; Spoerer et al., 2017). Very successful results were paid for by long computation times (de Santos-Sierra et al., 2015) (in the order of minutes per image) or sophisticated neuronal network software, running on large computer systems (Li et al., 2017). One simple segmentation strategy was the transformation of the neuronal network into a binary skeleton, a technique that we tested with SynoQuant's build in grayscale skeletonization algorithm and compared it to binary skeleton algorithm of “Ne.Mo” Software (Billeci et al., 2013). It worked well on the neurites but the cell bodies were also skeletonized in such a way that the cell somata were included in the segmented image (data not shown). Our approach was developed to detect neurite networks reliable without somata in a large number of images in a reasonable timeframe (less than 10 min for ~200 images, 640 × 480 pixel on an average laptop PC). The two algorithms were quite different because the RCS algorithm extracted rather thicker neurites while NeurSegm created an artificial skeleton of the neurite paths. As discussed before, both algorithms were sensitive to focus change, resulting in noise for time laps velocity measurements.

Computer based plasma membrane segmentation and automated detection of highly magnified cells was used since 20 years in many studies, mostly relaying on the contrast and brightness differences between membranes and substrate (Dimopoulos et al., 2014; Kaur and Sahambi, 2016; Meijering et al., 2016; Lee et al., 2018). The task appeared easy, when the image quality was good and the contrast high. However, in some images the membrane structures were only partially recognizable and some stretches of the cell perimeter faded into the background. Our algorithms could compensate for missing membrane pieces by employing a completion method, based on proximity of distinct membrane sections. The computational time per image were linear (slope = 2) correlated to the image size, e.g., 25 s for a 1,300 × 1,000, 12- s for a 900 × 700- and 4 s for a 640 × 480-pixel image.

The common difficulty of all segmentation algorithms were the occurrence of a flickering effect, when the segmented objects were viewed in time laps videos. The images are two-dimensional projections of cells, moving also very slightly up and down and causing thereby local focus variations. The resulting partial detection of the structures could be eliminated reliably by averaging successive image frames, however at the cost of time resolution loss. Image series that were filtered in this way could not be used for motility measurements, because the apparent velocity was rendered much lower than in reality. Therefore, it proved the best strategy to use the original segmented objects for motility analysis even at the cost of higher noise.

### Motility Measurement

Measuring the motility speed of individual structures in dense cellular networks, where individual cells were not readily discernable, is very challenging. Experiments on a long-term basis (48 h) were often problematic, because cell density and focus could change. Additionally, there were also limits that prevented even our program “SynoQuant” to conduct a meaningful measurement. These included general very low image contrast, very bad focus or very large images that took a long time to process. These parameters needed to fall into reasonable ranges. As a rule of thumb, the program worked well on gray scale image series that are subjectively sharp, and contrasted and not larger than 1,000 × 1,000 pixels.

The widely used method of individual particle tracking (IPT) could not address those issues. IPT had been implemented in many image analysis programs (Chenouard et al., 2014), but in our system it failed, because neighboring cells and other mobile objects (organelles, debris and neurites) were moving across or partially overlapped their structures, causing the tracking software to produce severe errors. We tried SynoSoft's particle tracker (data not shown) and could confirm that the automated tracking algorithm lost their targets, every time cells tightly touched each other. Manual optical tracking of individual cells produced very similar results as “TrackMate” (see Figure 7G) and “COPRAMove,” but was very time consuming. Therefore, so far, only cells without neurites or extended appendages that were plated at a sufficiently low density could be analyzed with IPT (Jaqaman et al., 2008). Our alternative approach was the pre-segmentation of cellular structures and subsequent global determination of general mean motility by either differential subtraction or correlation methods.

In order to test the robustness of our motility speed measurement algorithms, we used a “Cell simulator” software module to vary mutually interfering parameters. Our results showed that the “COPRAMove” algorithm was best suited for velocity determination, because it was very stable against variable cell densities, brightness and focus changes. Its core process was based on a Pearson's correlation coefficient between consecutive image frames that proved to be largely insensitive to brightness changes. An additional integration of a ratio component compensated a changing focus and neutralized highly variable structure densities (cells and neurites). Our results on simulated cells showed that the major sensitive parameter was the velocity of moving cells, while all other contributing factors played a minor role for the calculation of the velocity descriptor.

The algorithm “DiffMove” that focused on local brightness differences between frames, was also very effective, calculating the cellular velocity, when no other system components were changed. The idea of differential frame analysis was successfully tested on plasma membrane dynamics of single glia cells and produced a sensitive and effective motility index (Sild et al., 2013). However, the very common increase of cell and neurite densities in our system was very disturbing for this algorithm, leaving only experiments with constant focus, brightness and cell number for reliant motility speed analysis.

Another general problem, involving global average cell mobility measurement, was the lack of absolute values for motility speed (van Larebeke et al., 1992), which made it difficult to compare two or more independent data series that were obtained under different imaging conditions. In order to overcome this problem, we standardized images with respect to size, brightness, magnification and calibrated them according to an independent simulated dataset. This was achieved in SynoQuant by a “Cell simulator” module that simulated the velocity, the shape and the environment of cells. The artificial cells resembled the observed cells with respect to size, brightness and contrast. The simulation software moved them in a defined pattern with variable velocities and generated a linear velocity transfer function that was used to calculate an absolute velocity (e.g., in μm/frame) for real cells.

### Pericyte Plasma Membrane Motility

Besides the determination of global cell mobility in low magnification images, membrane contractility and intracellular structures could be quantified, if individual cells were separated and higher magnified. We studied the dynamics of plasma membranes in pericytes and measured the changes in membrane contraction and extension velocities. After exposure of pericytes to 1-h of OGD, all cells became more stationary and reduced their motility when measured with the “DiffMove” algorithm. Since we used isolated stationary single cells, for shorter times, the motility results matched the visual impressions very well, because the focus, brightness and cell density were kept constant. However, there were large individual differences in the relative ruffling velocity in between the single cells. This was not contributed to their variable size, but most likely to the unknown individual-specific metabolic status of the cells.

The ruffling of pericyte plasma membrane and their extension/contraction was monitored by measuring the perimeter length and the cell area. Since there were fast, apparently random, changes due to extension/contraction of the cell, it appeared not appropriate in our experimental system to merge these motility parameters into one single velocity descriptor, however, this might be dependent on the specific scientific question.

## Conclusion

General motility analysis of dense cellular preparations is a relatively new field in cell biology. We have found surprisingly little literature addressing this problem, although many methods are available for time lapse video analysis in the artificial intelligence field. In this study we have analyzed and tested two newly developed algorithms for general motility measurement in cell cultures and we introduced several image processing and segmentation algorithms. Our strategy was based on automated segmentation of individual structures and their subsequent dynamic quantification with “COPRAMove” and/or “DiffMove” velocity algorithms. The results show that “COPRAMove” is far more robust than “DiffMove” when the operator has to deal with unstable micro-environmental conditions, like variable focus, brightness- and density changes. “DiffMove” is faster and very precise in a clear defined, and well-structured situation, especially suited for isolated single cell analysis. Our SynoQuant software package could help to get access to the dynamic aspect of cellular morphometry.

## Author Contributions

AH created the software design, programmed the software, supervised the MSc student MA-Q and wrote the manuscript. LA-A performed pericyte experiments. MA-Q performed the hippocampal experiments. ZR supervised the MSc student LA-A, designed the pericyte experiments and participated in the software design.

### Conflict of Interest Statement

The authors declare that the research was conducted in the absence of any commercial or financial relationships that could be construed as a potential conflict of interest.
