# Supplementary material for: Quantitative Determination of Cellular-and Neurite Motility Speed in Dense Cell Cultures
Source: Front Neuroinform. 2019 Mar 12;13:15. doi: 10.3389/fninf.2019.00015 (PMC6423175; doi:10.3389/fninf.2019.00015)
Supplement: Supplementary file 6 [file Data_Sheet_1.PDF]

Supplemental videos

1. Supplmov_01.mp4
'COPRAmove' velocity analysis of intracellular organelle trafficking in a chicken telencephalon glia cell.

2. Supplmov_02.mp4
Pseudo-DIC 48-hour time laps video of neuronal network in hippocampal neuron-glia culture.

3. Supplmov_03.mp4
Dual motility analysis of a growing neuronal cellular network and microglia in a highly magnified cell hippocampal neuron-glia culture.

4. Supplmov_04.mp4
48-hour time laps video of colored overlay of segmented soma and neurite network in hippocampal cells.

5. Supplmov_05.mp4
'MoveDiff' membrane ruffling measurement of a contracting and expanding pericyte, recorded in a 9-hour time laps video.
